# Creating a nutritional traffic light able to help in education for diabetes self-management

**DOI:** 10.1186/1758-5996-7-S1-A172

**Published:** 2015-11-11

**Authors:** Priscila Nogueira Lara, Marcella Lobato Dias Consoli

**Affiliations:** 1 Santa Casa Belo Horizonte, Itauna, Brazil

## Background

Diabetes mellitus is a chronic disease that requires ongoing medical care, and education on self-management, in order to avoid acute complications and reduce the risk of chronic complications. Feeding recommendation for people with diabetes is no different from healthy people, being based on adequate intake of carbohydrates, proteins and fats adjusted to metabolic targets, energy needs and individual preferences. Educating and motivating people with diabetes to follow continuously eating plan is a major chronic challenge. Applying diabetes education through educational materials reinforcing the theory of good nutrition in order to facilitate the daily meal plan and food choices can be a strategy to improve adherence to nutritional therapy.

## Objective

To develop an educational material with nutritional information on food labeling that is able to assist the population with diabetes to make healthful choices.

## Materials and methods

The educational material was divided into four parts: 1- labeling food; 2- nutritional information; 3- complementary nutritional facts; 4- nutritional traffic light. The traffic light colour approach to nutritional signpost labelling requires criteria that define the green color if key nutrient is less than or equal 5% of recommendation, amber if between 5% and 25% and red if key nutrient is more than 25. The material was applied to individuals with type 2 diabetes and rated by a specific questionnaire in order to verify its effectiveness.

## Results

**Figure 1:**
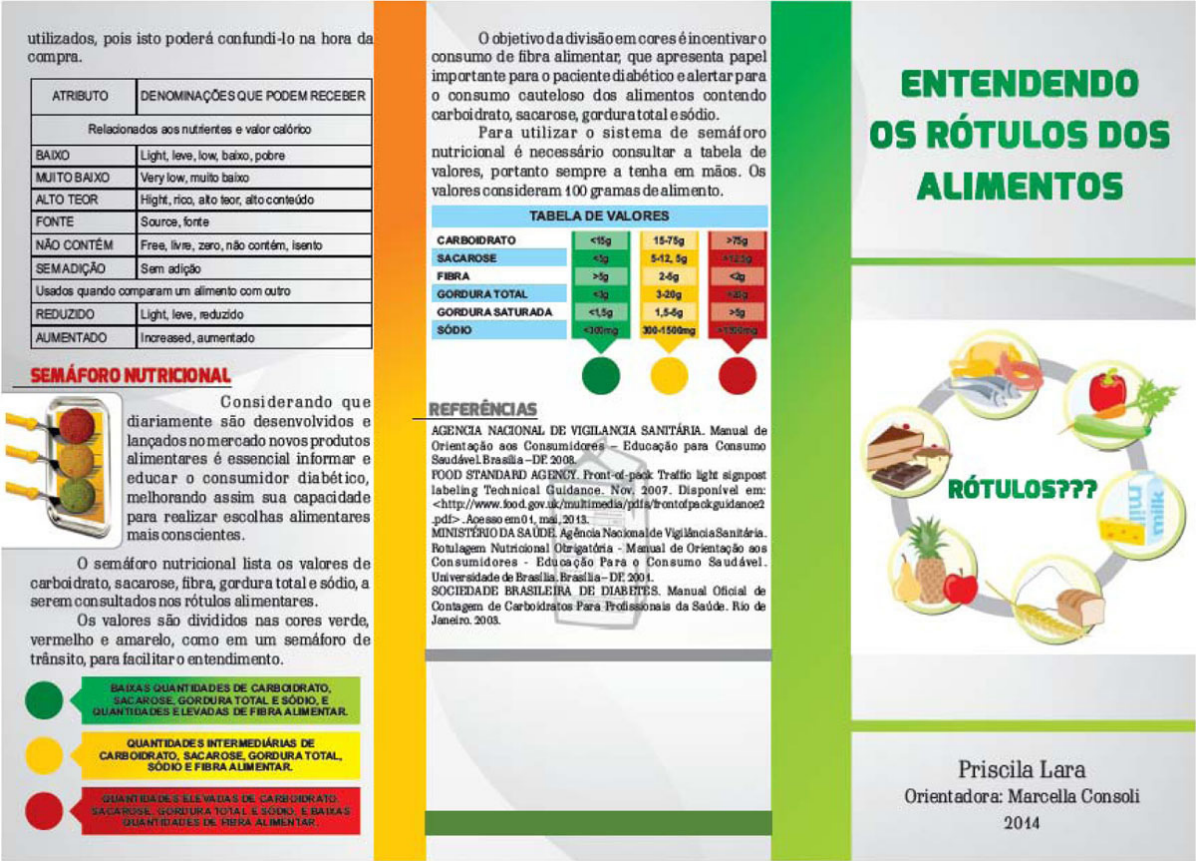
The educational pamphlet.

The chosen format was an informative pamphlet. The final document is located in Figure 1. The nutritional traffic light was able to make the nutritional labeling simpler and easier for the consumer's understanding. The use of traffic light colour approach in food labeling has already been tested in other studies that observed changes in the consumer choice behavior. On the evaluation of the effectiveness of the material, the seven specific questions related to the attributes of nutrition labeling, answered by 36 patients who completed the study, a significant increase in the percentage of hits in four of them. This average increase was 42,2%.

**Figure 2:**
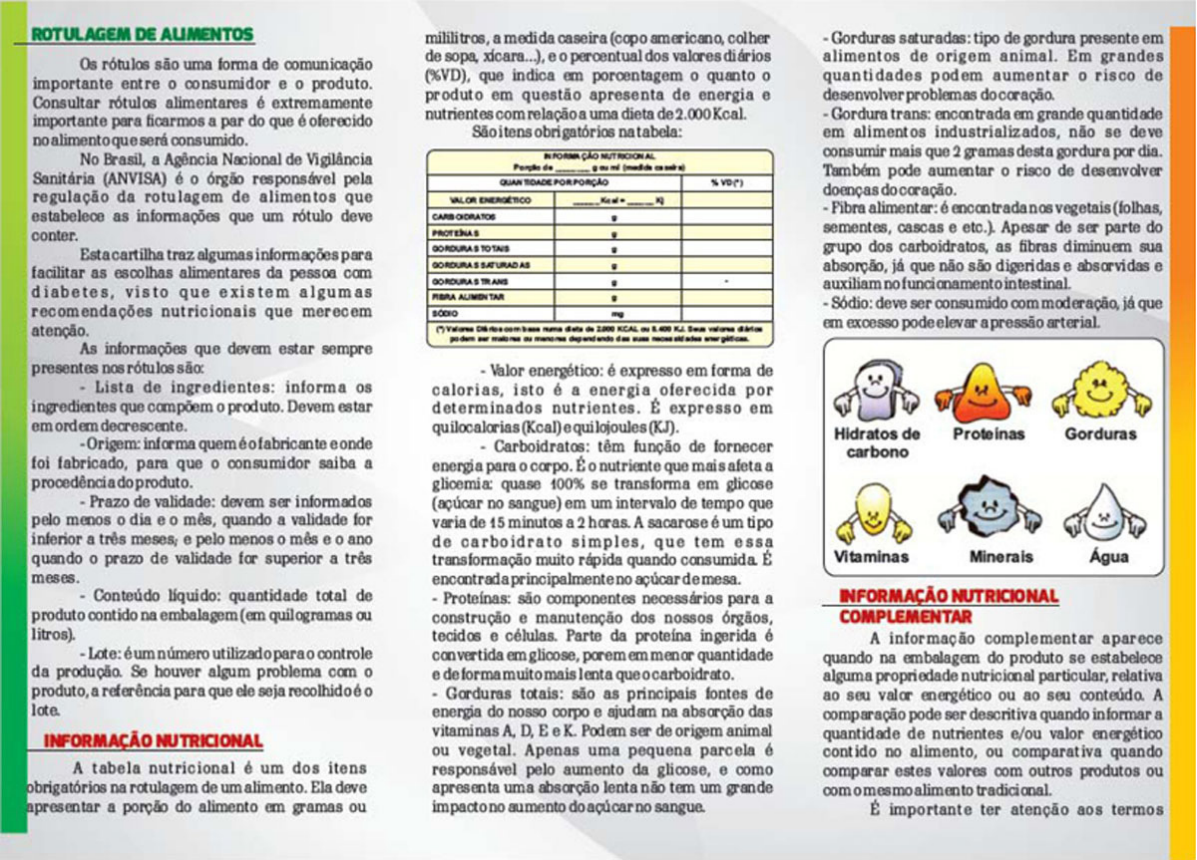
The educational pamphlet.

## Conclusion

The elaborate educational material was considered satisfactory and fulfilled its role of assisting the food choices of people with diabetes.

